# Study on Post-Treatment Relapse in HBeAg Positive CHB Patients

**DOI:** 10.1371/journal.pone.0141072

**Published:** 2015-11-02

**Authors:** Junfeng Lu, Jin’e Li, Yali Liu, Shan Ren, Zhenhuan Cao, Yi Jin, Lina Ma, Chengli Shen, Xinyue Chen

**Affiliations:** 1 Beijing You’an Hospital, Capital Medical University, Beijing, China; 2 Department of Infectious Diseases and Microbiology, Graduate School of Public Health, University of Pittsburgh, Pittsburgh, United States of America; University Hospital of Essen, GERMANY

## Abstract

**Background:**

Many factors are associated with post-treatment relapse in CHB patients, and there are no effective factors to predict relapse. In this study, we investigate the influence factors associated with post-treatment relapse and their predictive value in HBeAg positive CHB (eP-CHB).

**Methods:**

The factors associated with post-treatment relapse were analyzed firstly by a retrospective study in eP-CHB. Variables included age, sex, regimen, baseline HBeAg and HBV DNA level, total course of treatment as well as duration of consolidation therapy after HBeAg seroconversion. The predictive effects of the influence factors were evaluated in an eP-CHB prospective cohort.

**Results:**

89 patients were enrolled in the retrospective study, 42(47.2%) relapsed after discontinuation of treatment. Factors related to post-treatment relapse were total course of treatment, duration of consolidation therapy and baseline HBV DNA level. Relapse rates in patients with total course >36 months, consolidation duration >12 months and baseline HBV DNA level < 1.0E+5IU/ml were lower than those of total course <24 months (*P* = 0.002), consolidation duration≤12 months (*P* = 0.011) and baseline HBV DNA level > 1.0E+7IU/ml (*P* = 0.01) respectively. Patients with HBV DNA≥1.0E+7IU/ml plus HBeAg<200COI at baseline had the highest relapse rate and cumulative relapse rate than the other three arms (*P* = 0.048 and 0.008 respectively). Logistic regression analysis demonstrated that baseline HBV DNA level, duration of consolidation therapy and combination of baseline HBV DNA and HBeAg (Ig^DNA^/Ig^HBeAg^) were independent factors to predict post-treatment relapse. The model based on baseline Ig^DNA^/Ig^HBeAg^ and consolidation duration worked well in predicting post-treatment relapse in the prospective study and the accuracy, specificity, sensitivity, PPV and NPV for prediction were 80.3%, 81.1%, 79.2%, 73.1% and 85.7% respectively.

**Conclusions:**

Virological factors including baseline HBV DNA, HBeAg and treatment course were major influence factors associated with post-treatment relapse in eP-CHB. Patients with higher HBV DNA and lower HBeAg levels at baseline, shorter total course as well as consolidation therapy were more likely to develop relapse after discontinuation of therapy. The antiviral therapy in eP-CHB patients should be individually managed at different levels. It is better to treat those with higher viral load and lower HBeAg levels at baseline for a longer course, especially longer consolidation duration so as to decrease the relapse rate.

## Introduction

Antiviral therapy is crucial for chronic hepatitis B (CHB) patients. With the rising rate of patients withdrawing from antiviral treatment, a growing number of relapses are occurring. HBeAg seroconversion was a widely used treatment endpoint for HBeAg positive CHB (eP-CHB) [1–2]. However relapse is inevitable after discontinuing the antiviral agent. The relapse rates in eP-CHB patients who acquired HBeAg loss/seroconversion with consolidation therapy were approximately 40%-90% in nucleos(t)ide analogue (NA)-treated patients[3–5], and 30%-40% in interferonα(IFNα)-treated patients[6–7], which might vary in different studies. There are many factors influencing post-treatment relapse, including host factor, such as age[8–9], treatment factors, such as regimen and course of treatment[4,6,8], and viral factors such as genotype and viral load [10–11]. Reasons for the discrepancy may be due to different study type (retrospective or prospective) and criterion of drug withdrawal. Moreover, there is still lack of study on predictive effect of the influence factors by prospective study. Here, we designed a retrospective and a prospective study to explore the influencing factors of post-treatment relapse and their predictive effect in eP-CHB patients.

## Materials and Methods

### Subjects

#### Retrospective study

In retrospective study, eight-nine Chinese CHB patients were enrolled in Beijing You’an Hospital. The inclusion criteria were as follows: (1) Patients were diagnosed as eP-CHB according to China’s guideline of prevention and treatment for chronic hepatitis B (2010 version) with ALT>2ULN and HBV DNA>1.0E+4IU/ml; (2) IFNα(Peg IFNα or standard IFNα), NAs or combination of the both had been used for antiviral treatment; (3) Consolidation therapy was at least for 6 months after achieving HBV DNA<20IU/ml and HBeAg seroconversion; (4) Regular follow-up was conducted after drug withdrawal.

#### Prospective study

Sixty-one eP-CHB patients with ALT>2ULN and HBV DNA>1.0E+4IU/ml who were referred to Beijing You’an Hospital from January 2010 were recruited. The patients received IFNα (Peg IFNαor standard IFNα), NAs or combination of the both according to patient' s preference. Patients were excluded if (1) there was evidence of decompensated cirrhosis, either diagnosed or suspected HCC; (2) they were co-infected with HAV, HCV, HDV, HEV or HIV; (3) they accompanied with autoimmune hepatitis, alcoholic liver disease and inherited metabolic liver disease. Consolidation treatment was at least for 6 months in IFNα-treated and 1 year in NA-treated patients after achieving HBV DNA<20IU/ml and HBeAg seroconversion.

### Serological testing

Liver function, HBV markers and HBV DNA levels were conducted at an interval of 3 months from the start of treatment to the end of follow-up. Liver function was assayed by routine automated analysis system. HBV DNA levels were assayed using real-time PCR with the lower detection limit of 20 IU/ml (COBAS Taqman, Roche diagnostics). HBeAg was measured by the use of Elecsys MODULAR ANALYTICS E-170(Roche diagnostics).

### Clinical definitions

Virological response was defined as an undetectable serum HBV DNA level (<20IU/ml). Serological response was defined as HBeAg seroconversion. Relapse was defined as HBV DNA level >1.0E+3IU/ml in any two consecutive visits with or without HBeAg reccurence. Non-relapsers were followed-up to over 6 months. Patients would withdraw from the study once relapse were confirmed.

### Statistical analysis

The analyses were performed using the Statistical Package for the Social Sciences (SPSS), version 16.0. Normally distributed data were displayed as mean±SD, while non-normally distributed data were displayed as medium(range). Student *t* tests and Wilcoxon's Sign rank tests were used to perform grouped pair-wise comparison. Enumeration data were compared usingχ^2^ tests. Potentially related factors were also tested by univariate and multivariate logistic regression analysis. The Kaplan-Meier method was used to calculate the cumulative relapse rate. Receiver operating characteristic curve (ROC curve) was used to analysis the relevant predictive factor for relapse. Differences were considered significant at *P*<0.05 level.

### Ethics

The study was approved by the Human Ethics Committee of Beijing Youan Hospital, Capital Medical University, Beijing, China. Informed written consent was obtained from each patient in the study.

## Results

### Factors associated with post-treatment relapse in retrospective study

#### Demographic characteristics

A total of 89 patients aging from 16–63 years were enrolled in the study. Among them, 61 were male, 28 were female, 27 were NA-treated and 62 were IFN-treated (including IFNαplus NA and IFNαmonotherapy). The total treatment course ranged from 12 to 58 months with a median of 26 months and the period of followed-up ranged from 6 to 43 months with a median of 11 months. 47.2% (42/89) relapsed after discontinuation of treatment. Sex, age, total course, consolidation therapy, baseline HBV DNA and HBeAg level are no significantly different between NA-treated and IFN-treated patients.

#### Relevant factors for post-treatment relapse

All possible factors associated with post-treatment relapse were listed in Table 1. Firstly, stratified analysis revealed that total course, consolidation treatment and baseline HBV DNA level were associated with relapse. The longer the total course and consolidation treatment were, the lower the rate of relapse was. Relapse rates in patients with total course >36 months, consolidation therapy >12 months were lower than those of total course <24 months (23.1% vs 63.2%, *P* = 0.002) and consolidation therapy≤12 months (25.0% vs 55.4%, *P* = 0.011) respectively. Relapse rate was higher in patients with baseline HBV DNA level>1.0E+07 IU/ml than those with <1.0E+05IU/ml (59.0% VS 22.2%, *P* = 0.01). Patients with low baseline HBeAg level tend to have higher relapse rate than those with high HBeAg level, but no significant difference was detected. Patients with high HBV DNA plus low HBeAg levels at baseline had significantly higher relapse rate compared with other HBV DNA and HBeAg combination groups (*P* = 0.048). However, there were no significant differences in relapse rate between patients with different age, sex and regimens. In addition, the factors related to relapse for NA and IFN treatment regimen were evaluated separately (S1 Table). Consistent with the combined analysis results, the longer total treatment course and longer consolidation treatment were associated with low relapse rate and high HBV DNA plus low HBeAg levels was associated with high relapse rate in both NA and IFN treatment groups. Because the sample size was relatively small, we did not do the formal statistical comparison.

**Table 1:** Possible factors associated with post-treatment relapse.

factor	N	Relapse (*n*)	Mean±SD	χ^2^ /t	*P*
Sex				0.01	0.922
Male	61	29			
Female	28	13			
Age				-1.493	0.193
relapser	42		36.6±10.0		
Non-relapser	47		33.1±11.9		
Regimen				2.265	0.132
IFN-treated	62	26			
NA-treated	27	16			
Total course (months)				9.961	0.007
<24	38	24			
24~36	25	12			
>36	26	6			
Consolidation therapy (months)				6.493	0.011
≤12	65	36			
>12	24	6			
Baseline HBV DNA level (IU/ml)				6.677	0.035
<1.0E+05	18	4			
1.0E+05~1.0E+07	32	15			
>1.0E+07	39	23			
Baseline HBeAg level(COI)				1.960	0.375
<200	46	25			
200~1000	20	8			
>1000	23	9			
Baseline HBV DNA(IU/ml) plus HBeAg(COI)				7.886	0.048
<1.0E+07, ≥200	21	7			
<1.0E+07, <200	29	12			
≥1.0E+07, ≥200	22	10			
≥1.0E+07, <200	17	13			

Secondly, the seven factors above were individually introduced in univariate logistic regression model. Serum HBeAg and HBV DNA levels were log10 transformed for analysis. Only consolidation duration and baseline HBV DNA level (Ig^DNA^) were statistically significant. Although there was no statistical difference, baseline HBeAg level was negatively related to relapse. Based on the consideration for viral replication and protein expression, we introduced the concept of Ig^DNA^/Ig^HBeAg^ in univariate regression model, which was referred to a combined marker for baseline HBeAg and HBV DNA level. The result indicated that this factor was statistically significant (Table 2). Further, Ig^DNA^/Ig^HBeAg^ together with other factors was introduced in multivariate logistic regression model, showing that consolidation therapy and Ig^DNA^/Ig^HBeAg^ were significantly correlated with relapse (Table 3).

**Table 2:** Factors associated with post-treatment relapse in univariate logistic regression model.

Factor	*B*	*SE*	*Wald*	*P*	Exp(B)
Consolidation duration	-.063	.025	6.519	.011	.939
Ig^DNA^	.479	.182	6.928	.008	1.615
Ig^DNA^/Ig^HBeAg^	.452	.164	7.636	.006	1.571

**Table 3:** Factors associated with post-treatment relapse in multivariate logistic regression model.

Factor	*B*	*SE*	*Wald*	*P*	Exp(B)
Consolidation duration	-.080	.032	6.382	.012	.923
Ig^DNA^/Ig^HBeAg^	.518	.185	7.859	.005	1.679

#### Cumulative relapse rate

**Fig 1:**
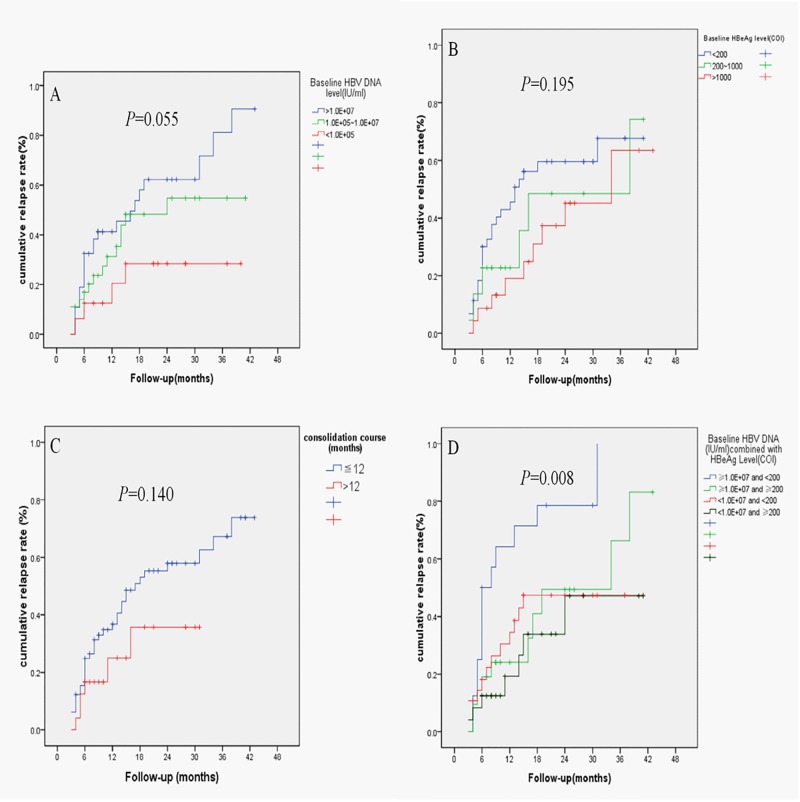
Cumulative relapse rate among subgroups with different baseline factors. Cumulative relapse rates in patients with baseline HBV DNA level > 1.0E+7IU/ml were higher than HBV DNA level between 1.0E+5 IU/ml and 1.0E+7 IU/ml, and HBV DNA level < 1.0E+5IU/ml (A), with baseline HBeAg level < 200COI higher than HBeAg level between 200COI and 1000COI, and HBeAg>1000COI (B), consolidation treatment > 12 months lower than ≤ 12 months group (C), and HBV DNA level ≥ 1.0E+07IU/ml plus HBeAg level < 200COI at baseline higher than the other three groups with statistically significant differences (D).

As displayed in Fig 1A, 1B and 1C, cumulative relapse rates in patients with high baseline HBV DNA, low baseline HBeAg and consolidation treatment ≤12 months were higher than those with low HBV DNA, high HBeAg and consolidation therapy >12 months, but the differences were not statistically significant between them. However, patients with high HBV DNA level (≥1.0E+07IU/ml) plus low HBeAg level (<200COI) at baseline had the highest cumulative relapse rate among all the four arms, as shown in Fig 1D (*P* = 0.008).

### Assessment of the predictive value of the relevant factors with relapse by the prospective study

#### Demographic characteristics

A total of 61 eP-CHB patients aging from 17–61years were enrolled in the prospective study and followed up over 6 months. Among them, 31 were male, 30 were female, 38 were IFN-treated (including IFNαplus NA and IFNαmonotherapy) and 23 were NA-treated. The mean age was 31.8±8.82 years. The median course of treatment was 25(18–48) months and the median consolidation duration was 15(6–30) months. 39.3% (24/61) relapsed after discontinuation of treatment. Also, there were no statistical differences in the sex, age, total course, consolidation therapy, baseline HBV DNA and HBeAg level between IFN-treated and NA-treated groups.

#### Prediction of post-treatment relapse

**Fig 2:**
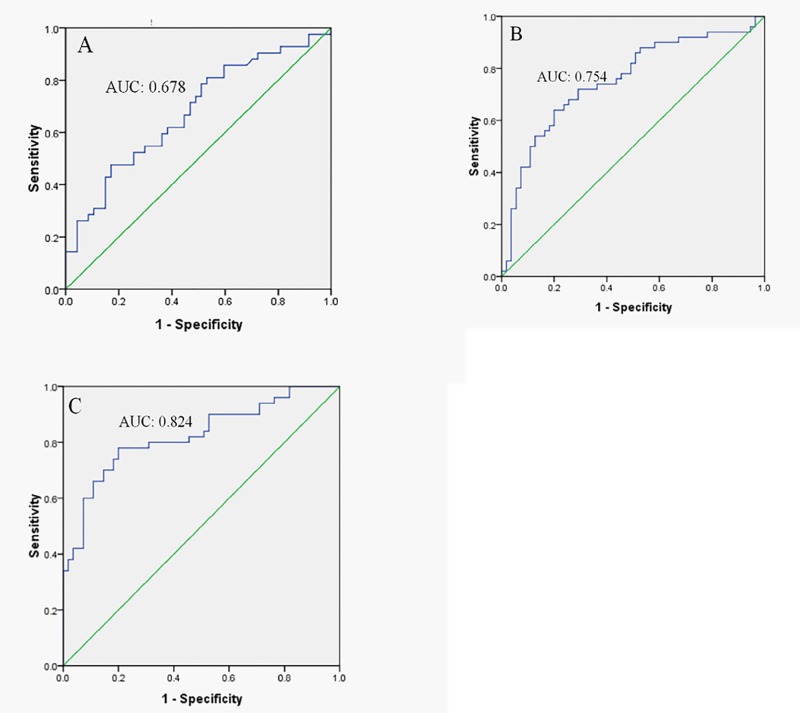
ROC curves of the independent factors based on retrospective study for predicting relapse. ROC curve of the Ig^DNA^ in differentiating post-treatment relapse with AUC 0.678 (A), ROC curve of Ig^DNA^/Ig^HBeAg^ with AUC 0.754 (B) and Ig^DNA^/Ig^HBeAg^ combined with consolidation duration with AUC 0.824 (C).

Based on retrospective data, Baseline Ig^DNA^, baseline Ig^DNA^/Ig^HBeAg^ and duration of consolidation therapy were three independent factors influencing post-treatment relapse. Subsequently, ROC curves of the Ig^DNA^, Ig^DNA^/Ig^HBeAg^ and Ig^DNA^/Ig^HBeAg^ combined with consolidation duration in differentiating relapse were drawn. Area under the curves (AUC) was 0.678, 0.754 and 0.824 respectively (Fig 2A, 2B and 2C). The Ig^DNA^/Ig^HBeAg^ combined with consolidation duration had the biggest AUC, which had the most significant value in predicting relapse. Date from the prospective study was introduced in the ROC generated by Ig^DNA^/Ig^HBeAg^ combined with consolidation duration. The optimized cut-off value of 0.590 produced a sensitivity of 79.2%, specificity of 81.1%, positive predictive value (PPV) of 73.1%, negative predictive value (NPV) of 85.7%, possibility of correct classification of 80.3% (Table 4). The results support that baseline Ig^DNA^/Ig^HBeAg^ combined with consolidation duration are good prediction markers for post-treatment relapse.

**Table 4:** The predictive effect of baseline Ig^DNA^/Ig^HBeAg^ combined with consolidation duration in prospective study.

Factor	Predictive outcome	Observed relapse	Observed no relapse	sesitivity	specificity	PPV	NPV	accuracy
Ig^DNA^/Ig^HBeAg^ combined with consolidation duration				79.2%	81.1%	73.1%	85.7%	80.3%
	relapse	19	7					
	no relapse	5	30					

## Discussion

In recent years, post-treatment relapse has become the biggest challenge in clinical practice over the world. Identifying the most efficient predictive factors for post-treatment relapse may be a promising way to improve antiviral efficacy and reduce relapse.

In our study, we analyzed the possible factors associated with post-treatment relapse in eP-CHB in the retrospective study. The results showed that total course of antiviral treatment, duration of consolidation treatment after HBeAg seroconversion, baseline HBV DNA and baseline Ig^DNA^/Ig^HBeAg^ were related to post-treatment relapse, but age, sex and regimens had no relationship with relapse.

Patients with longer total course (>36 months) and consolidation duration (>12 months) were less likely to relapse after discontinuation of treatment. The latter was an independent predictive factor for relapse, which was reported in previous studies. In the study from Pan, a total of 136 NA-treated eP-CHB patients achieving treatment endpoints were enrolled. It was reported that those with duration of consolidation therapy >11 months had lower relapse rate than those with consolidation duration≤11 months (26.0% VS 58.7%)[12]. In another study on Peg IFNαin eP-CHB, extended course of treatment was associated with high sustained response rate and lower relapse rate[13]. As recommended in Chinese CHB guideline[14], consolidation treatment for over 1 year and total treatment course for over 2 years are needed before discontinuing NAs for eP-CHB patients achieving complete response, and the recommended treatment course of Peg IFNαis only 1 year. In our study, 23.1% patients relapsed with a total course of more than 36 months, and 25.0% relapsed with consolidation duration of more than 12months. Thus, it is difficult to define a certain total course and consolidation treatment. We consider that the antiviral treatment for eP-CHB patients should be individually optimized with set goals and different course. Total course, especially consolidation duration after HBeAg seroconversion should be extended under the premise of safety and effectiveness to achieve better treatment outcomes.

In our study, high HBV DNA level at baseline was associated with high relapse rate and cumulative relapse rate. High baseline HBV DNA was an independent risk factor for post-treatment relapse, which was consistent with the findings of the previous study on eP-CHB patients treated with LAM [11,15]. The possible mechanism includes: 1) HBV DNA level is positively correlated with intrahepatic cccDNA level and higher HBV DNA reflects the greater amount of intrahepatic cccDNA storage, which is the source of relapse;2) High HBV DNA level is indicative of active viral replication and insufficient host immunity for viral clearance. The implementation of antiviral therapy at the moment increased the risk of drug resistance. It is recommended by both CHB guidelines of EASL[1] and AASLD[16] that NA with potent antiviral efficacy and low risk of resistance should be used. In the Chinese experts’ recommendation, initial combination therapy should be considered to lower the risk of post-treatment relapse in patients with high viral load.

Baseline HBeAg level has long been recognized to be associated with HBeAg seroclearence with IFN [17] or NAs [18]treatment in eP-CHB. The lower HBeAg level at baseline was, the more likely HBeAg seroclearence could be achieved and vice versa. In our study, we were astounded to discover that low HBeAg level at baseline was associated with high post-treatment relapse. Usually, low viral load produced low HBeAg level indicative of balance between viral replication and protein expression. However, in our study, HBV DNA level seems to be not consistent with HBeAg level. Therefore, we performed a analysis in patients with different baseline HBV DNA and HBeAg levels. The results showed that patients with HBV DNA≥1.0E+07IU/ml plus HBeAg<200COI had the highest relapse and cumulative relapse rates among all the four groups. Ig^DNA^/Ig^HBeAg^ defined as HBV DNA combined with HBeAg level and better reflecting the baseline virological characteristics was demonstrated to be an independent predictive factor for post-treatment relapse. Higher HBV DNA level and lower HBeAg level were associated with greater risk of relapse. The reason may be due to the mutations at pre-C/BCP in HBV genome, which is a common strategy for HBV to escape from host immune pressure.

In the latter part of this study, we drawn three ROC curves based on the results of the retrospective study and tested the predictive effect in the prospective study. AUC from baseline Ig^DNA^/Ig^HBeAg^ plus consolidation duration was greater than that from baseline Ig^DNA^/Ig^HBeAg^ and baseline HBV DNA level alone, which had a sensitivity of 79.2%, specificity of 81.1%, PPV of 73.1%, NPV of 85.7% in predicting relapse in prospective study. This indicated that it would be better to take both virological factors and treatment factors into account in prediction of post-treatment relapse. In this study, NPV which predicted the probability of not developing relapse after drug withdrawal provided a basis that partial patients could discontinue treatment in limited treatment course and avoid adverse events from extended course of treatment and waste of medical resource. On the other hand, PPV can be used to find patients with high risk of relapse, preventing relapse by extending the treatment course. Among the three factors, baseline Ig^DNA^/Ig^HBeAg^ is related to viral replication and viral expression. However, consolidation duration is more essential factor predictive of post-treatment relapse, which is not only the predictive factor of relapse but also the method of reducing relapse by extending the consolidation duration.

In summary, for eP-CHB patients, low HBeAg level and high HBV DNA level at baseline are associated with high post-treatment relapse rate. Patients with longer total course of treatment as well as consolidation treatment have lower post-treatment relapse rate. Baseline Ig^DNA^/Ig^HBeAg^ combined with duration of consolidation therapy has a good predictive value of post treatment relapse. We hereby suggest that antiviral therapy for eP-CHB patients should be individualized upon baseline HBeAg and HBV DNA levels and for those with high viral load and low HBeAg levels, extending treatment course and duration of consolidation treatment after HBeAg seroconversion should be considered.

## Supporting Information

S1 Table Possible Factors Associated with Post-treatment Relapse by NA and IFN Treatment. (DOCX)
