# Supplementary material for: Study on Post-Treatment Relapse in HBeAg Positive CHB Patients
Source: PLoS One. 2015 Nov 2;10(11):e0141072. doi: 10.1371/journal.pone.0141072 (PMC4629894; doi:10.1371/journal.pone.0141072)
Supplement: S1 Table — (DOCX) [file pone.0141072.s001.docx]

|  | factors | | N | Relapse | | Mean±SD |
| --- | --- | --- | --- | --- | --- | --- |
|  |  |  |  | n | % | / |
| NA-treated | Sex | male | 16 | 10 | 62.5 | / |
|  |  | female | 11 | 6 | 54.5 |  |
|  | Age | Relapse | 16 | / | / | 41.5±8.587 |
|  |  | Non-relapser | 11 | / | / | 35.8±10.932 |
|  | Total course (months) | <24 | 14 | 12 | 85.7 | / |
|  |  | 24～36 | 7 | 3 | 42.9 |  |
|  |  | >36 | 6 | 1 | 16.7 |  |
|  | Consolidation  therapy (months) | ≤12 | 20 | 15 | 75.0 | / |
|  |  | >12 | 7 | 1 | 14.3 |  |
|  | Baseline HBV DNA level (IU/ml) | <1.0E+05 | 6 | 1 | 16.7 | / |
|  |  | 1.0E+05～1.0E+07 | 10 | 6 | 60.0 |  |
|  |  | >1.0E+07 | 11 | 6 | 54.5 |  |
|  | Baseline HBeAg level(COI) | <200 | 12 | 8 | 66.7 | / |
|  |  | 200～1000 | 10 | 5 | 50.0 |  |
|  |  | >1000 | 5 | 3 | 60.0 |  |
|  | Baseline HBV DNA(IU/ml) plus HBeAg(COI) | <1.0E+07，≥200 | 7 | 4 | 57.1 | / |
|  |  | <1.0E+07，<200 | 9 | 6 | 66.7 |  |
|  |  | ≥1.0E+07，≥200 | 8 | 4 | 50.0 |  |
|  |  | ≥1.0E+07，<200 | 3 | 2 | 66.7 |  |

**Supplemental Table 1: Possible Factors Associated with Post-treatment Relapse by NA and IFN Treatment**

| IFN-treated | Sex | male | 45 | 18 | 40.0 | / |
| --- | --- | --- | --- | --- | --- | --- |
|  |  | female | 17 | 8 | 47.1 |  |
|  | Age | Relapse | 26 | / | / | 33.3±10.003 |
|  |  | Non-relapser | 36 | / | / | 32.4±12.194 |
|  | Total course (months) | <24 | 24 | 12 | 50.0 | / |
|  |  | 24～36 | 18 | 8 | 44.4 |  |
|  |  | >36 | 20 | 6 | 30.0 |  |
|  | Consolidation  therapy (months) | ≤12 | 45 | 21 | 46.7 | / |
|  |  | >12 | 17 | 5 | 29.4 |  |
|  | Baseline HBV DNA level (IU/ml) | <1.0E+05 | 12 | 3 | 25.0 | / |
|  |  | 1.0E+05～1.0E+07 | 22 | 9 | 40.9 |  |
|  |  | >1.0E+07 | 28 | 17 | 60.7 |  |
|  | Baseline HBeAg level(COI) | <200 | 34 | 17 | 50.0 | / |
|  |  | 200～1000 | 10 | 3 | 30.0 |  |
|  |  | >1000 | 18 | 6 | 33.3 |  |
|  | Baseline HBV DNA(IU/ml) plus HBeAg(COI) | <1.0E+07，≥200 | 14 | 3 | 21.4 | / |
|  |  | <1.0E+07，<200 | 20 | 6 | 30.0 |  |
|  |  | ≥1.0E+07，≥200 | 14 | 6 | 42.9 |  |
|  |  | ≥1.0E+07，<200 | 14 | 11 | 78.6 |  |
